# Amniotic Membrane as a Main Component in Treatments Supporting Healing and Patch Grafts in Corneal Melting and Perforations

**DOI:** 10.1155/2020/4238919

**Published:** 2020-02-14

**Authors:** Katarzyna Krysik, Dariusz Dobrowolski, Edward Wylegala, Anita Lyssek-Boron

**Affiliations:** ^1^Department of Ophthalmology with Pediatric Unit, St Barbara 5th Regional Hospital, Trauma Centre Medykow Square 1, 41200 Sosnowiec, Poland; ^2^Chair and Clinical Department of Ophthalmology, School of Medicine, Dentistry Division in Zabrze, Medical University of Silesia, Panewnicka 65 Str., 40760 Katowice, Poland; ^3^Department of Ophthalmology, District Railway Hospital, Panewnicka 65, 40-760 Katowice, Poland

## Abstract

**Purpose:**

To report on surgical approaches using amniotic membrane applications and patch grafts in corneal melting and perforations. Anatomical and functional results, including advantages and disadvantages of the interventions, will also be explored.

**Methods:**

A five-year retrospective analysis of 189 surgical treatments involving corneal melting with perforation was performed. In one evaluated treatment type, a graft of amniotic membrane, often folded one to three times, was sutured with the epithelial side facing the previously mechanically debrided corneal tissue. A larger monolayer amniotic patch was then sutured, with the epithelial side facing the top of the first membrane, to the perilimbal conjunctiva. For corneal patch grafts, the size-fitting technique of graft trephination was applied, and the donor-recipient junctions were sewn with interrupted sutures. All the procedures were evaluated, noting outcomes and complications of surgery, preoperative and postoperative visual acuities, postoperative intraocular pressures, graft rejection, and other late comorbidities and complications.

**Results:**

We performed 119 amniotic membrane applications (63%) and 70 corneal patch grafts (37%). Anatomical reconstruction of the anterior chamber was achieved in 157 eyes, of which 102 eyes (86%) received an amniotic membrane and 55 eyes (79%) were treated with the patch graft technique. In 63 eyes (33%), more than one amnion or graft treatment was necessary to close the corneal perforation.

**Conclusions:**

The success of medical and surgical management depends on the cause of corneal melting, and amniotic membrane applications often require further intervention; nevertheless, patch grafts deliver better tectonic reconstruction than amniotic membrane alone.

## 1. Introduction

Serious progressive corneal stromal dissolution may lead to corneal ulceration and, subsequently, corneal perforation. Corneal perforation is always a medical emergency requiring rapid medical intervention. There are several known predisposing factors leading to serious stromal dissolution and disintegrity of the globe. These include infection, Sjögren syndrome, systemic disease, sterile inflammation, Stevens–Johnson syndrome, tear-film abnormality, prior ocular surgery, collagen vascular disease, neurotrophic keratopathy, persistent epithelial defect, chemical or thermal injury of the ocular surface, and use of topical medications that contain preservatives [1–9].

A superfamily of proteases called metzincins (zinc-dependent metallopeptidases), which include the matrix metalloproteinase (MMP) family, are well-known regulators of extracellular matrix proteolysis in the microenvironment. Metalloproteinases play a fundamental role in the inflammatory response. Their proteolytic activity can regulate pro-and anti-inflammatory cascades and are also regulative in the processes of tissue remodelling. The MMPs are shown to have a role in infectious and noninfectious causes of corneal tissue destruction [9–11]. The management and timing of corneal melting, ulceration, and consecutive perforation depends on the cause, size, corneal infiltration, location of perforation, and status of other ocular tissues [12, 13]. Nonsurgical treatments include reducing inflammation, addressing the underlying cause, using anticollagenases, treating coexistent infections, prescribing antiglaucoma treatments, and optimising epithelial healing (using bandage soft contact lenses, autologous serum eye drops, or lacrimal punctal occlusions). The diagnosis of corneal perforation can sometimes be difficult or even impossible as complex interactions exist between the different components of the ocular surface [1, 2, 14]. A wide range of conditions can, therefore, result in similar functional effects at the ocular surface. The key to prompt diagnosis and treatment of corneal melting and perforation is in cooperation with other medical specialists, such as rheumatologists, immunologists, and microbiologists [15]. If pharmacological treatments fail or are insufficient, surgery becomes the only possibility for restoring the structural integrity of the globe; it may also serve as an adjunct for another definitive treatment method. Surgical treatments for corneal perforation include tarsorrhaphy, tissue adhesives, corneal transplants, conjunctival flaps, tenon-derived patch grafts, amniotic membrane transplants (AMT), and therapeutic ptosis with botulinum toxin [1, 3, 7, 16]. The surgical treatment of corneal perforation usually begins with a tectonic treatment to stabilise the eye. Optical surgery is then performed to restore visual function [2, 17].

The amniotic membrane (AM), the innermost layer of a foetal membrane, synthesises a variety of biological factors, including growth factors, cytokines and neurotrophins; it has many important biological properties in the context of corneal surgery. The AM enhances epithelialisation by facilitating the migration of epithelial cells, reinforcing adhesion of basal epithelial cells, promoting epithelial differentiation to maintain epithelial phenotype, and modulating the proliferation of normal corneal, conjunctival and limbal fibroblasts [18–20]. AM also prevents apoptosis and has antimicrobial properties and inhibits scarring, inflammation, and neovascularisation [18–20]. The advantage of AMT is that the cornea exhibits negligible immunological responses against AM, even though fibroblasts, mesenchymal cells, and the amniotic epithelium express HLA I and HLA II [21].

Small- or medium-sized perforations, leaking descemetoceles, and corneal melting may be treated with AMTs, tectonic corneal patch grafting (PG), or anterior lamellar keratoplasties. Sclerokeratoplasty, penetrating keratoplasty (PK), and total penetrating keratoplasty are reserved for large perforations with excessive melting, tissue destruction, and extensive necrosis [2–4, 9, 12, 21].

The aim of this study is to report on patients who underwent surgical treatment for corneal perforation using AMT and PG methods. We report on the surgical techniques, complications of treatment, and anatomical and functional results in this group of patients.

## 2. Materials and Methods

This study is a five-year (2014–2018) retrospective review of a case series of 189 eyes with severe ocular surface disorders resulting in corneal melting with perforation. All cases were from the Ophthalmology Department of Saint Barbara Hospital Trauma Centre, Sosnowiec, Poland. The analysed data from the medical records included demographic information; medical histories; preoperative and postoperative best spectacle-corrected visual acuities (BSCVA) measured using the Snellen visual acuity (VA) chart; details, outcome, and complications of surgery; postoperative intraocular pressures; graft rejection and other comorbidities and complications; and results of accessory examinations (e.g., microbial tests). All patients signed an informed consent form before any surgical procedure. In accordance with the Polish law, this retrospective observational study does not require the approval of a local bioethical committee.

All patients were diagnosed with corneal melting and perforation after a complete slit-lamp ocular examination with B-mode ocular ultrasonography and swept-source anterior segment optical coherence tomography (Casia SS-1000, Tomey, Nagoya, Japan). Patients were treated with one of the following two surgical treatment methods: AMT or PG. The crucial aim of surgery was to restore the integrity of the globe. The choice of the surgical technique was dependent on size, extent, depth of corneal melting, and state of the other internal ocular tissues. For the study, we qualified perforations that had a limited necrotic zone after the initial anti-infective treatment which did not require radical treatment (total and full-thickness therapeutic transplantation). When anterior chamber (AC) structures were intact and only the shallowness of AC was observed, AMT was performed. The amniotic membrane was generally proposed as the first line of treatment. The approach was chosen based on the availability of the tissue, ease of application, and the possibility of replacing it with the tectonic graft. On contrary, in eyes that had the iris or lens embedded in the corneal perforation, corneal PG was the method of choice for surgical treatment. All surgeries were performed as quickly as possible under topical or general anaesthesia.

AMs were obtained from a tissue bank (FRK Homograft, Zabrze, Poland). The membranes were taken during caesarean sections, washed with phosphate-buffered saline (PBS) and 50 IU/mL penicillin, and stored at −80°C in Dulbecco's modified Eagle's medium (DMEM) and glycerol (1 : 1). Immediately before use, the tissues were defrosted, washed from the cryoprotective medium with PBS, and cut into appropriate sizes that matched the size of the corneal tissue damage. All steps were performed under aseptic conditions. A combined surgical approach was applied: the inner membrane, serving as the graft and folded 1 to 3 times, was sutured with the epithelial side facing the previously mechanically debrided corneal tissue. The larger monolayer amniotic patch was sutured, with the epithelial side facing the top of the first membrane, to the perilimbal conjunctiva. AMs were sewn with 10–0 nylon-interrupted sutures. A bandage contact lens was placed on the graft. Four weeks after surgery, when amnion transplant was integrated with the bed, the bandage contact lens was removed, and the sutures were taken out.

The donor corneas for PG were originated from our own hospital or cooperative tissue banks. The freehand technique of graft trephination was applied for corneal patch grafts. We used microtrephines of 3 to 6 mm diameter, and the graft oversize was 0.75–1 mm. Each fundus of the ulcer was cleaned from necrotic tissue around the perforation. The ulcer border surrounding epithelium was also gently removed. The graft was shaped to match the host bed; if necessary, its round shape was modified with scissors for a better fit. Finally, the donor-recipient junction was sewn using 10–0 nylon-interrupted sutures with the assistance of a viscoelastic substance in the AC. Surgical host-bed preparation with the removal of necrotic tissue was performed in all cases.

Simultaneously with surgical treatment, intensive pharmacological topical treatments were performed and consisted of the following components: steroids, antiviral gels, lubricants (including autologous serum), broad-spectrum antibiotic, antimycotic, or antiprotozoal drops. As well, general steroids, antivirals, antibiotics, antimycotics, and immunosuppressive agents in cases of autoimmune origin (e.g., azathioprine and mofetil mycophenolate) were used.

Because of the complex nature of underlying diseases, the individual approaches were confirmed by microbiologists and rheumatologists and tailored to the specific needs of each patient. Additionally, the prolonged administration of immunosuppression necessitated medical controls and consultations with nephrologists, immunologists, and hepatologists. Modifications were applied according to the progress of recovery. Topical antimicrobial therapy was routinely applied for 21 days, with extensions if necessary. Steroid doses were lowered (at monthly intervals) and tapered off before the sixth month of therapy. In cases of unstable epithelia, persistent intensive lubrication was administered hourly. If necessary, patients were also treated with cycloplegics and antiglaucoma medications.

All patients were admitted for 1–3 days after operation and were followed up every 2 weeks for a period of 2 months, monthly for a minimum of 6 months, and at differing intervals thereafter. The mean follow-up was 36 months.

The XLSTAT-Biomed (Addinsoft SARL, France) software was used for statistical analysis and to calculate means and standard deviations. Analysis of variance was used to compare the baseline characteristics and postoperative outcomes among subgroups of patients who received surgical treatment for corneal perforation. A *p* value of <0.05 was considered statistically significant.

## 3. Results

Between January 1, 2014, and December 31, 2018, 189 eyes of 183 patients with corneal melting and perforation were operated on with either the AMT or PG technique. This patient group consisted of 85 females (87 eyes), whose mean age was 61.42 ± 15.18 (range 21–84 years), and 98 males (102 eyes), whose mean age was 57.74 ± 15.98 (range 18–85 years). There was no statistically significant difference with respect to gender and age between both groups (*p* > 0.05). In 6 patients (2 females and 4 males), both eyes required a one-time surgical treatment. Due to bilateral corneal perforation, one female and three males with chemical alkali burns and one female and one male with immune disorders, respectively, underwent simultaneous bilateral AMT.

In the whole study group, 189 surgical tectonic procedures, comprising 119 AMTs (63%) and 70 corneal PGs (37%), were performed. In the female group of patients (87 eyes), the surgical procedures consisted of 56 AMTs (64%) and 31 corneal PGs (36%). In the male group of patients (102 eyes), the procedures consisted of 63 AMTs (62%) and 39 corneal PGs (38%).

We recognized the following reasons of corneal melting in our study group: chemical burns (82%), infectious keratitis (34%), sterile inflammation (27%), neurotrophic corneal ulcers (loss of the neurosensory innervation of the cornea) (22%), surgical injury (18%), and ocular trauma (17%). The dominant infectious factor was bacterial infection (67%).

Inflammatory factors, consisting mainly of autoimmune diseases, led us to recognize the coexistence of rheumatoid arthritis (74%), ankylosing spondylitis (18%), and lupus erythematosus (8%). Eye injuries in the group were dominated by chemical (mainly alkali) burns of the ocular surface in 26 eyes (79%). The surgical procedures on the cornea that lead to corneal melting with consecutive perforation were refractive surgery (4 eyes) and phacoemulsification and trabeculectomy (1 eye). A detailed list of indications for each surgery type is presented in Table 1.

For large, full-thickness stromal involvement with corneal melting, AMT was the preferred surgical treatment method. However, in eyes with active inflammation and abundant purulent discharge, AMT had limited utility. PG was chosen for local, peripheral, or paracentral corneal disorders, often with involvement of the other ocular internal tissues such as the lens, iris, or vitreous, and in cases where these tissues had direct contact with the site of corneal perforation.

Pharmacological treatment after AMT and PG consisted of topical antimicrobial agents (antibiotics, aminoglycosides, fluconazole, and voriconazole) for five times daily, steroids (dexamethasone) for five times daily, and lubricants hourly. Steroids, general antibiotics/antimycotics/antiviral agents and immunosuppressive agents (azathioprine, cyclosporin A and mofetil mycophenolate) were administered individually according to the specific needs of the patient. Prescriptions depended on the melting aetiology and were based on consultations with microbiologists and rheumatologists. Cycloplegics and antiglaucoma medications were administered, if necessary.

Final anatomical reconstruction of the AC and ocular integrity was achieved in 157 eyes, of which 102 eyes (86%) underwent AMT and 55 eyes (79%) were treated with the PG technique.

More than one AMT or PG treatment was necessary to close the corneal perforation in 63 eyes (33%). In 36 eyes (30%), re-AMT was performed, and in 7 other eyes (5.9%), there were 3 AMT surgeries: 3 eyes postcorneal refractive surgery, 3 eyes with neurotrophic ulceration, and 1 eye of a patient with immunodeficiency. Additionally, 5 eyes that had undergone PG (7%) required subsequent AMT to enhance epithelialisation.

Twenty-three eyes (19.3%) that underwent AMT and 13 eyes (18.6%) that underwent PG required penetrating keratoplasty after a mean of nine months due to the expansion of the corneal tissue melting and necrosis. This group consisted of 23 eyes with confirmed infections (16 bacterial, 4 viral, and 3 fungal infections), 11 eyes with rheumatoid arthritis, and 3 eyes with neurotrophic corneal ulceration. Penetrating keratoplasties were performed with large graft oversize after exact necrotic tissue removal.

To improve the optical results of the tectonic treatment, successive surgical procedures were necessary, as reported in Table 2.

Secondary posterior chamber intraocular lens (PCIOL) implantation consisted of three surgical methods: in-the-bag implantation, in-the-sulcus implantation, and transscleral fixation. The time of the successive optical surgery ranged from 6 to 24 months after the final tectonic treatment.

In patients with autoimmune diseases, loosening of the corneal sutures was a predisposing factor for melting. This was reported in 16 eyes (23%) from the PG group and warranted immediate suture removal and increased intensity of anti-inflammatory treatment.

The main aim of the tectonic treatment was to achieve a watertight ocular surface with a continuous epithelial layer. Persistent epithelial defects refractory to medical therapy were the most common complication of surgical treatment and occurred in 104 eyes. Because of decreased corneal sensitivity, this complication was reported mainly in neurotrophic (26 eyes) and autoimmunological patients (33 eyes). Additionally, abnormal tear production was predominant in these groups of patients. Intensive medical therapy was initiated to increase ocular surface moisture with preservative-free lubricants, and cyclosporine A drops and drops containing 20% autologous serum. To limit existing tear evaporation, constant eye patches, partial tarsorrhaphies, bandage soft contact lenses, or ptosis with botulinum toxin were applied. As an adjunctive therapy, anticollagenases, vitamin C (to facilitate collagen synthesis), and oral doxycycline (100 mg applied twice daily for 6 to 8 weeks) were prescribed.

Consequent glaucoma or ocular hypertension was reported in 39 eyes (21%), which included 14 eyes after AMT (11.8%) and 25 eyes after PG (35.7%), respectively. Medical treatment comprised one or two topical agents. Refractory cases required surgical intervention; trabeculectomy was performed in 10 eyes (25.6%), and transscleral cyclophotocoagulation was performed in 2 eyes (5.1%).

BSCVA before surgical treatment ranged from light perception to 0.1. Postsurgical improvement of visual acuity was limited because of the nature of underlying disease and surgical technique, particularly that of AMT. BSCVA improvement was observed in 14 eyes (11.8%) from the AMT group and 15 eyes (21.4%) from the PG group, ranging from light perception to 0.4.

Despite repeated surgical tectonic procedures and intensive pharmacological treatment with the assistance of other specialists, 18 eyes (9.5%) with corneal melting and perforation were lost. This included 5 eyes (2.6%) which developed endophthalmitis and required evisceration despite intensive antimicrobial treatment. Loss of light perception was reported in 7 eyes from the study group. This resulted from persistent hypotony in 4 eyes (2.1%) and retinal detachment in 3 eyes (1.6%).

## 4. Discussion

The management of corneal lacerations with melting and consequent perforation depends on their size, location, and underlying disease. It is nonetheless important to consider the surgeon's experience and the availability of donor corneal tissue [3–5, 13, 19, 20]. When donor corneas are unavailable, either AMs should be considered, or temporary or permanent conjunctival flaps are used to restore ocular surface integrity [3].

In the absence of a definite diagnosis, ocular surface diseases can usually be managed effectively, provided that the approach and therapy is chosen based on the severity of the observed functional effects. It is therefore important to have a systematic approach regarding this decision [15].

Namba et al. [17] suggested that AMT is sufficient in maintaining corneal shape, although it is not able to provide tectonic integrity. They also recommended against multilayered AMTs for permanent wound closure in corneal perforations involving the optical axis because the central cornea can become cloudy, with the final visual result requiring optical keratoplasty. Multilayered AMTs are a useful tool in the management of corneal ulceration or perforation to restore corneal integrity and to minimise the risk of corneal grafts [13, 17, 19]. In our series, we also preferred AMTs because of the lower risk of complications and their ability to promote epithelial healing at the core of the corneal melting. Chan et al. [19] recommended multilayered AMT (the “Swiss Roll” technique) in cases of severe thinning and localised perforation to limit the need for a corneal graft. However, in small corneal tissue deficits, it can be difficult to fill and stabilise the many slippery layers of the lower AM. Regardless of the chosen technique, the proper diagnosis of patients and the selection of experienced surgeons will increase the chances of success in this method of treatment.

Uhlig and Müller [22] proposed fixation of the resorbable sutures of multilayered AMTs to the cornea. In their opinion, it is a useful option to stabilise the ocular surfaces with sterile, large, deep, and perforating corneal lacerations, but this requires prospective investigations. In our practice, we normally use nonresorbable nylon sutures to avoid immunological reactions to the sewing material.

AMs simplify the restoration of the corneal epithelium and recovery of the ocular surface because of their anti-infectious, anti-inflammatory, antiangiogenic, and immunomodulatory properties [4, 8, 14, 23]. They are also nonantigenic and cause less astigmatism when compared to peripheral PGs; moreover, in PGs, postoperative graft-host interface hazing or sutures may interfere with the visual axis [2, 14].

AMs contain various protease inhibitors that inhibit and prevent corneal melting. When used for perforations and deep stromal lacerations, they suppress inflammation and promote epithelialisation. The various inhibitory and proinflammatory cytokines and other molecules identified in AMs have contradictory and opposing actions. For example, IL-6 and IL-8 are proinflammatory MMPs that promote vascularisation and melting. However, tissue inhibitors of MMPs have the opposite role of inhibiting both actions [18]. Complications from AMT are rare and often reflect the progression of the underlying aetiology [19]. AMs should be avoided in cases of active infection as they impede AC and fundus visualisation. Our observations and practices are consistent with that of other authors [1, 24, 25]. In cases of active infection, we prefer to perform PG after total removal of the infected and damaged corneal tissues. However, the severity, aetiology, and progression of infection determine the timing and modification of treatment plans. Whenever possible in this study group, we applied intensive topical and general antimicrobial and anti-inflammatory treatments as a first line of therapy, while delaying surgery to a later date.

The results in our AMT group are comparable with those achieved by Ozdemir et al. [4]. However, the mean period between failed AMTs and subsequent PKs was shorter in our study group. This may be the result of better corneal tissue availability from our own tissue bank and a wider range of primary indications for surgery.

In the study of Bouazza et al. [3], the main purpose of surgical treatment was to restore ocular integrity in nontraumatic corneal perforations. The predominant aetiologies of corneal perforations in this study, as well as those of our series, were corneal infection and inflammation. Their results of tectonic treatment with AMTs and PGs are convergent with ours.

Corneal perforations are usually smaller than the corneal melting area. Thus, grafted tissue should be larger than the primary observed lesion, after considering the amount of necrotic tissue removed. Corneal or sclerocorneal patches are best used in cases with peripheral corneal perforations and descemetoceles. They restore the stable watertight ocular surface and promote faster visual rehabilitation [2, 5, 11]. Corneal PG, preceded by removal of the frequently-infected necrotic tissue, decreases the risk of infection spreading into the internal tissues of the eye. However, there is also the risk of rejection of this tissue, resulting from the preservation of donor endothelium [14].

The outcome of surgical treatment of corneal melting with perforation depends not only on surgical technique but also on pathogenic mechanisms and perioperative immune status. Thus, control of corneal melting and surface infection are critically important for graft survival [2, 4, 8, 15].

To achieve a satisfactory improvement in visual acuity, multistage surgical approaches are often necessary [6]. The most frequent procedure in our study group was PK alone or with simultaneous cataract extraction and PCIOL implantation. Both methods were common in eyes primarily treated with AMT: 42.6% versus 33.3% and 27.9% versus 22.2%, respectively. It was the result of central corneal scars resulting from keratitis, the nature of transplanted tissue (cloudy and opaque AM), and repetitive tectonic corneal surgery. Additionally, cataract extraction with PCIOL implantation was frequently performed in both groups of patients. Pars plana vitrectomy with cataract surgery, alone or with PCIOL implantation, was performed in eyes with prior intensive intrabulbar inflammation.

## 5. Conclusions

The successful medical and surgical management of corneal melting with consequent perforation requires recognition of the primary disease. Initial or parallel treatment of the underlying condition strongly influences the final results. Autoimmune cases require special attention; interdisciplinary treatment may be helpful, but ophthalmologists should not be afraid to use immunosuppressive drugs. According to our results and data from cited papers, primary surgery should be minimalistic, and attention should be paid to the preparation of the graft bed, whether it is AM or PG.

Many data indicate that, apart from specific situations, the use of the amniotic membrane should be the preferred method of choice. Use of AM gives time for pharmacological treatment, protects exposed tissues, and does not preclude transplantation. PG is a radical treatment, but more secure than primary total keratoplasty. It is an adequate treatment in cases of anterior chamber tissue exposure. Total grafts should be applied only in advanced necrosis or in keratitis that is unresponsive to pharmacological treatment [26, 27]. Because of the heterogeneity of the causes of corneal melting, there is no single universal treatment method for corneal perforation.

## Figures and Tables

**Table 1:** Patient characteristics by surgical technique and medical indication.

Characteristics	Total (*n* = 189) *N* (%)	Female (*n* = 87) *N* (%)	Male (*n* = 102) *N* (%)
Amniotic membrane transplantation	**119 (63.0)**	**56 (47.1)**	**63 (52.9)**
Indication for surgery			
Infection	29 (15.34)	16 (18.39)	13 (12.75)
Inflammation	29 (15.34)	17 (19.54)	12 (11.76)
Neurotrophic	35 (18.52)	16 (18.39)	19 (18.63)
Ocular trauma	26 (13.76)	7 (8.05)	19 (18.63)

Corneoscleral patch graft	**70 (37.0)**	**31 (44.3)**	**39 (55.7)**
Indication for surgery			
Infection	36 (19.05)	16 (18.39)	20 (19.61)
Inflammation	21 (11.11)	10 (11.49)	11 (10.78)
Neurotrophic	6 (3.18)	3 (3.45)	3 (2.94)
Ocular trauma	7 (3.7)	2 (2.3)	5 (4.9)

**Table 2:** Successive optical surgical treatment after primary tectonic surgery.

Surgical technique	Total (*n* = 115) *N* (%)	AMT group (*n* = 61) *N* (%)	PG group (*n* = 54) *N* (%)
PK	44 (38.3)	26 (42.6)	18 (33.3)
PK with cataract surgery and PCIOL implantation	29 (25.2)	17 (27.9)	12 (22.2)
Cataract surgery with PCIOL implantation	21 (18.3)	9 (14.8)	12 (22.2)
Secondary implantation of PCIOL	3 (2.6)	1 (1.6)	2 (3.7)
Iridoplasty	12 (10.4)	5 (8.2)	7 (13.0)
Pars plana vitrectomy	3 (2.6)	1 (1.6)	2 (3.7)
Pars plana vitrectomy with cataract surgery with PCIOL implantation	3 (2.6)	2 (3.3)	1 (1.9)

## Data Availability

The patient data used to for this study are included within the article.
